# Clathrin-mediated post-fusion membrane retrieval influences the exocytic mode of endothelial Weibel-Palade bodies

**DOI:** 10.1242/jcs.200840

**Published:** 2017-08-01

**Authors:** Nicola L. Stevenson, Ian J. White, Jessica J. McCormack, Christopher Robinson, Daniel F. Cutler, Thomas D. Nightingale

**Affiliations:** 1 MRC Cell Biology Unit, Laboratory of Molecular Cell Biology, University College London, Gower Street, London, WC1E 6BT, UK; 2 Centre for Microvascular Research, William Harvey Research Institute, Barts and The London School of Medicine and Dentistry, Queen Mary University of London, Charterhouse Square, London EC1M 6BQ, UK

**Keywords:** Endocytosis, Endothelium, Weibel-Palade bodies, Exocytosis, Clathrin, Dynamin

## Abstract

Weibel-Palade bodies (WPBs), the storage organelles of endothelial cells, are essential to normal haemostatic and inflammatory responses. Their major constituent protein is von Willebrand factor (VWF) which, following stimulation with secretagogues, is released into the blood vessel lumen as large platelet-catching strings. This exocytosis changes the protein composition of the cell surface and also results in a net increase in the amount of plasma membrane. Compensatory endocytosis is thought to limit changes in cell size and retrieve fusion machinery and other misplaced integral membrane proteins following exocytosis; however, little is known about the extent, timing, mechanism and precise function of compensatory endocytosis in endothelial cells. Using biochemical assays, live-cell imaging and correlative spinning-disk microscopy and transmission electron microscopy assays we provide the first in-depth high-resolution characterisation of this process. We provide a model of compensatory endocytosis based on rapid clathrin- and dynamin-mediated retrieval. Inhibition of this process results in a change of exocytic mode: WPBs then fuse with previously fused WPBs rather than the plasma membrane, leading, in turn, to the formation of structurally impaired tangled VWF strings.

This article has an associated First Person interview with the first authors of the paper.

## Abstract (precis)

**Summary:** Compensatory endocytosis plays key roles in Weibel-Palade body exocytosis. Inhibition of this process results in a change of exocytic mode and the release of von Willebrand factor as tangled strings.

## INTRODUCTION

Many cell types utilise regulated secretion as a means to release premade bioactive material from membranous carriers at the cell surface (Burgoyne and Morgan, 2003). These include soluble factors for release into the extracellular milieu, as well as integral membrane proteins that are then displayed for interaction with their cognate ligands. This is absolutely essential for a number of key physiological processes, including cell-to-cell communication, immune cell function, digestion, inflammation and haemostasis (Burgoyne and Morgan, 2003). The rate and frequency of carrier fusion with the plasma membrane can vary widely, with the fastest occurring during neurotransmitter release and the slowest during surfactant release from lamellar bodies in pneumocytes (Nightingale et al., 2012; Thorn et al., 2016).

Secondary to content release, exocytosis of secretory vesicles causes a net increase in the amount of membrane present at the cell surface. This is particularly apparent in cells with very large granules or cells that undergo rapid release (Thorn et al., 2016; Barg and Machado, 2008; Soykan et al., 2016). Compensatory endocytosis provides a means to limit this membrane expansion to maintain cell size and membrane tension, as well as to return key integral membrane proteins back into the cell (Gordon and Cousin, 2016; Reider and Wendland, 2011). This retrieval process is best understood in neurons and neuroendocrine cells where a number of discrete mechanisms occur. In neurons, the mode used depends on the stimulus received and includes full fusion (i.e. collapse) followed by clathrin-mediated endocytosis at a separate site, ‘kiss-and-run’ exocytosis (whereby a transient fusion event occurs and the granule reseals before collapse), clathrin-independent ultrafast endocytosis (UFE) and activity-dependent bulk endocytosis (Soykan et al., 2016; Okamoto et al., 2016; Watanabe et al., 2013, 2014). During neurotransmission, efficient and sustained neurotransmitter release is absolutely dependent on reformation of synaptic vesicles via clathrin-mediated endocytosis (CME) (Gordon and Cousin, 2016), either directly from the cell surface or from endosomes generated by other endocytic routes (Soykan et al., 2016). This is a tightly regulated process whereby membrane and specific cargo such as VAMP-2, synaptotagmin-1 and synaptophysin are retrieved in the appropriate ratio to allow reformation of a new synaptic vesicle (Soykan et al., 2016). During normal physiological stimulation this requires a number of adaptor proteins including AP-2, stonin-2 and AP180 (Willox and Royle, 2012). Retrieval in neuroendocrine cells is different; granules do not fully collapse and flatten out at the plasma membrane. Instead, they maintain their shape and are re-internalised intact from the plasma membrane in a process referred to as cavicapture (Henkel and Almers, 1996) or ‘fuse-pinch-linger’ (Ryan, 2003). These recaptured carriers can then either re-fuse with the plasma membrane at a later stage (if they still contain some cargo) or can be refilled with small transmitter molecules such as amines (Barg and Machado, 2008). This process requires dynamin (Henkel and Almers, 1996; Artalejo et al., 1995; Holroyd et al., 2002) and calcium (Henkel and Almers, 1996), and in some cases allows differential release of cargo based on the size of the fusion pore (Barg and Machado, 2008).

The purpose and mechanism of compensatory endocytosis in non-neuronal cell types is poorly characterised, especially in the case of the regulated secretory organelle of endothelial cells, the Weibel-Palade body (WPB). WPBs contain haemostatic and inflammatory mediators to be released into the vascular lumen (Lenting et al., 2015; Nightingale and Cutler, 2013; Valentijn and Eikenboom, 2013). The most abundant, and perhaps the most important, WPB cargo is von Willebrand factor (VWF), a 220 kDa glycoprotein that acts as a multifunctional mechanosensitive binding platform for blood components such as platelets. Following synthesis, this protein dimerises in the endoplasmic reticulum before transferring to the Golgi. Once in the low-pH environment of the trans-Golgi network (TGN) it forms a bouquet structure that stacks into extended coiled tubules. This process, alongside physical constraints imposed by the Golgi complex itself (Ferraro et al., 2016), confers a unique rod shape and a remarkable length (up to 5 µm) to the organelle (Valentijn and Eikenboom, 2013; Springer, 2014). Furin-mediated cleavage of the pro-peptide and formation of long disulphide-bonded concatemers is also initiated at the TGN and continues during organelle maturation.

Following exocytosis into the pH-neutral environment of the blood, and with the help of shear force generated by flow, VWF tubules unfurl and associate to form large platelet-catching strings (Dong et al., 2002; Erent et al., 2007). VWF extrusion is further expedited by some agonists that trigger the formation of a contractile actomyosin ring around the fused WPB membrane (Han et al., 2017; Nightingale et al., 2011). Mutations in the *VWF* gene leading to reduced protein expression, loss of or alterations to its binding sites, or a failure to form concatamers cause von Willebrand disease, the most common inherited bleeding disorder (Valentijn and Eikenboom, 2013; James and Lillicrap, 2013). WPBs also contain reservoirs of other proteins such as the type-1 integral membrane protein P-selectin, which is trafficked to the plasma membrane to recruit leukocytes in the first step of the leukocyte adhesion cascade (Bonfanti et al., 1989; McEver et al., 1989).

To our knowledge, few publications have addressed the mechanism of post-exocytic membrane recapture in endothelial cells and none of them in any detail (Valentijn et al., 2010; Zupancic et al., 2002). In 2002, Zupancic et al. confirmed that full fusion of WPBs results in a marked increase in membrane capacitance of 2.5-9.0 fF. This is followed by similar-sized stepwise reductions in membrane capacitance that most likely represent bulk retrieval of membrane (Zupancic et al., 2002). It would therefore appear that at least a proportion of compensatory endocytosis in endothelial cells results from the ‘en bloc’ internalisation of fused exocytic structures. Some, but not all of these events may represent longer-lived ‘lingering kiss’ exocytic events where a smaller 12 nm pore forms and eventually reseals following WPB fusion. This is thought to be the case for 10% of exocytic events during strong stimulation (Babich et al., 2008). Clathrin-coated pits, which may represent compensatory endocytic structures have also been noted on large secretory pod-like structures which are thought to result from intracellular fusion of WPBs (Valentijn et al., 2010). Whether these form before or after WPB fusion with the plasma membrane is unresolved (Valentijn et al., 2010; Mourik et al., 2013).

It is unclear whether compensatory endocytosis in endothelial cells serves a purpose beyond retrieval of membrane. WPBs by necessity must form at the TGN to allow normal release of functional strings (Lui-Roberts et al., 2005; Michaux et al., 2006); once exposed to pH 7.4 and unfurled, the VWF cannot be refolded. Compensatory endocytosis following VWF release thus cannot lead to the regeneration of functional granules for re-use as in neuroendocrine or neuronal cells (Gordon and Cousin, 2016). It is also unlikely to be required for retrieval of known integral membrane cargoes such as P-selectin and CD63 as these rapidly diffuse away from the WPB fusion site and can be retrieved through general endocytic pathways (Arribas and Cutler, 2000; Babich et al., 2009). Finally, if the purpose of WPB compensatory endocytosis is solely to retrieve membrane then this could be carried out anywhere on the plasma membrane and begs the question as to why clathrin-coated pits are found on fused structures containing VWF.

To address these issues, we investigated this process in human umbilical vein endothelial cells (HUVECs) using biochemical assays, transmission electron microscopy (TEM) and correlative live-cell imaging and TEM to define the extent, mode, mechanism and function of compensatory endocytosis. We demonstrate that changes in compensatory endocytosis affect the exocytic mode of WPBs.

## RESULTS

### A biochemical assay for monitoring compensatory endocytosis

Throughout this study, we use PMA as the stimulus for exocytosis for a number of reasons. Firstly, there are a large number of secretagogues that stimulate WPB exocytosis (more than 30) – some of which trigger a Ca^2+^-dependent release and some of which act via cAMP (Rondaij et al., 2006) – and PMA uses both; we wanted to monitor the effect on endocytosis irrespective of the route of stimulation. Indeed during physiological stimulation, endothelial cells are likely to be stimulated by multiple secretagogues at once and this often has a synergistic effect on release (Zografou et al., 2012). Secondly, later in this study we use a number of approaches to limit content release and endocytosis and as such it is important to use a secretagogue that will be unperturbed by such manipulations. As PMA is a lipid, it does not require binding of cell surface receptors for its action. Therefore, when we analyse results, we can exclude effects of pH on receptor ligand binding (e.g. histamine activates endothelial cells less efficiently at low pH; Babich et al., 2009). Similarly, we can exclude effects due to changes in receptor downregulation (as might occur during inhibition of endocytosis). Thirdly, phorbol 12-myristate 13-acetate (PMA) provides a strong stimulation and this makes monitoring its effect on endocytosis by biochemical or electron microscopy approaches as unequivocal as possible.

**Fig. 1.**
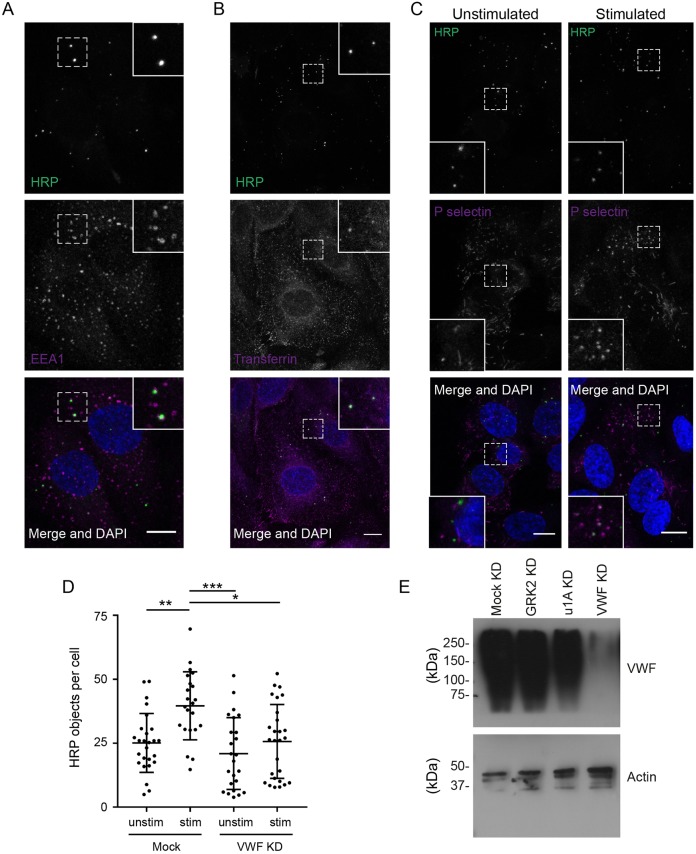
**Analysis of the extent of WPB-specific compensatory endocytosis.** (A) HUVECs were fed for 15 min with soluble HRP then fixed and stained for HRP (green) and EEA-1 (magenta). The nucleus is labelled with DAPI (blue). HRP and EEA1 co-localise, as indicated by white pixels, demonstrating HRP is within endosomes. (B) HUVECs were incubated with Alexa Fluor 568-tagged transferrin (magenta) for 2 min, washed and then uptake chased for 15 min in the presence of HRP (green). HRP and transferrin co-localise. (C) HUVECs fed with HRP (green) in the absence (left) or presence (right) of 100 ng/ml PMA for 10 min were fixed and stained for P-selectin. In unstimulated cells, P-selectin is stored within rod-shaped WPBs and does not localise with HRP. Following stimulation, P-selectin is present on round endocytic vesicles positive for HRP, demonstrating that it has been retrieved from the cell surface. Images in A-C are maximum intensity projections and boxed areas contain zooms of the regions of interest denoted by dashed outline. Scale bars: 20 μm. (D) Cells transfected with control or VWF siRNA were incubated with HRP with (stim) or without (unstim) 100 ng/ml PMA for 15 min and then treated as above. A 70% increase in HRP-positive objects is seen upon stimulation in control but not VWF knockdown cells (*n*=3). Error bars represent s.e.m. *P≤0.05, **P≤0.01, ***P≤0.001, Student's *t*-test. (E) Mock-treated and cells depleted for GRK2, the AP-1 subunit μ1A (u1A) or VWF were lysed and the levels of VWF and actin determined by SDS-PAGE and western blot. GRK-2 is required for controlling regulation of GPCR signalling whilst the AP-1 subunit μ1A is required for normal WPB formation. Only depletion of VWF itself affects total VWF levels.

To characterise the extent of compensatory endocytosis in endothelial cells, we began by comparing the rate of incorporation of fluid-phase markers into unstimulated and PMA-stimulated HUVECs. An assay monitoring horseradish peroxidase (HRP) uptake provided the most robust data, probably because even small amounts of HRP can be detected with great sensitivity using the tyramide signal amplification (TSA) system. After 15 min of uptake, HRP can generally be found in early endocytic organelles, as shown by partial co-localisation with EEA-1, an early endosomal marker (Fig. 1A). Similarly, transferrin internalised for 2 min and then chased for 15 min parallels the endocytic trafficking of the HRP (Fig. 1B). To show that HRP internalisation follows the fate of WPB components after exocytosis, we looked at its co-localisation with P-selectin, a WPB cargo. In unstimulated cells, P-selectin localised to rod-shaped structures throughout the cytosol and did not exhibit any significant co-localisation with HRP. However, following PMA stimulation, P-selectin appeared in small surface patches as well as in intracellular punctate structures that frequently, but not always, co-localised with HRP and were therefore endocytic (Fig. 1C) (Table S1). Importantly, treating HUVECs with PMA or a cocktail of histamine, adrenaline and IBMX for 15 min to stimulate WPB exocytosis resulted in a significant and reproducible increase in the amount of internalised HRP (Fig. 1D, Fig. S1A) suggesting that endocytosis was upregulated. To determine the proportion of this increase that was dependent on the exocytosis of WPBs, and to confirm that it was not an off-target effect of the secretagogue treatment, we knocked down VWF and measured HRP uptake in depleted cells. Knockdown (KD) cells lack VWF (Fig. 1E) and recognisable WPBs, and show a negligible increase in HRP incorporation following stimulation compared with control cells (Fig. 1D). From this, we can conclude that almost one-third of HRP incorporation post-stimulation labels compensatory endocytic structures.

We did attempt to use live-cell imaging to monitor compensatory uptake in real-time by following the incorporation of lipophilic dyes FM1-43 and FM4-64 into fused WPBs (visualised by the loss of GFP-tagged VWF). This proved difficult to characterise and quantify because of the high background noted both with spinning-disk and point-scanning confocal microscopy. Functional exocytic events (that release VWF strings into the blood flow) occur at the apical surface and this precluded our use of approaches such as TIRF microscopy. We did have some limited success by assaying the live incorporation of 10 kDa Dextran-tetramethyl-Rhodamine into fully fused WPBs, but again it was difficult to use this approach robustly as it labelled such vast numbers of structures with such a high background of fluorescence that quantification was inconsistent. Preliminary evidence from this work did, however, indicate that fusion of the WPB at the cell surface creates a compartment open to fluid-phase markers, which at least in the few instances we characterised, did not collapse into the membrane during the time of imaging (Fig. S1B).

### Ultrastructural time-resolved analysis of compensatory endocytosis

**Fig. 2.**
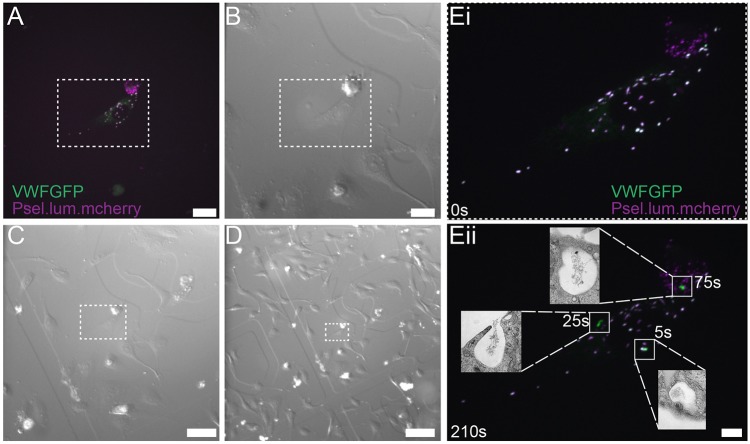
**Correlative live-cell imaging and electron microscopy to monitor kinetics and ultrastructure of compensatory endocytosis.** HUVECs were transfected with mCherry–P-selectinLum (magenta) and GFP-VWF and plated on gridded coverslips. (A-D) Fluorescent cells were identified (A; maximum intensity projection is shown) and DIC images acquired at (B) 40× (C) 20× and (D) 10× magnification to define localisation in *x* and *y* planes as denoted by the boxed region. Transfected cells were then stimulated with 100 ng/ml PMA and the same cell imaged by spinning-disc microscopy before fixation at arbitrary time points. (E) Images show the cell at (i) 0 s and (ii) 210 s. Fused WPBs release the mCherry (magenta) marker at the point of fusion and then more gradually release GFP-VWF during content release. Inserts show TEM images of the specific exit sites indicated. Time points shown represent time between loss of fusion marker and point of fixation. Scale bars: 20 μm (A,B), 50 μm (C), 100 μm (D) and 10 μm (E).

To characterise the route of compensatory endocytosis, as well as the timing and localisation of such events, we exploited a previously published assay to monitor the ultrastructure of exocytic sites in a time-resolved manner (Nightingale et al., 2011). We co-transfected endothelial cells with a GFP-tagged version of VWF and a truncated version of P-selectin lacking the transmembrane domain and cytoplasmic tail (mCherry–P-selectinLum). The former construct allows monitoring of VWF content release while the latter allows precise timing of the point of WPB fusion as it diffuses away rapidly in the medium (Nightingale et al., 2011). Cells were plated on gridded coverslips to allow precise localisation (Fig. 2A-D). Following stimulation with PMA, we imaged transfected cells using high-speed spinning-disk confocal microscopy and added fix at arbitrary time points (Fig. 2E). We then correlated the light microscopy with an EM analysis (correlated light and electron microscopy, CLEM) using serial sections. By monitoring the loss of mCherry–P-selectinLum we could precisely define how long from the point of fusion each exocytic event was fixed and thus correlate any compensatory endocytic structures seen by EM with time post fusion (Fig. 2Eii). All events characterised by this approach are full fusion events as the mCherry–P-selectinLum construct is too big to be released by the 12 nm pore reported as present during lingering kiss fusion (Babich et al., 2008).

**Fig. 3.**
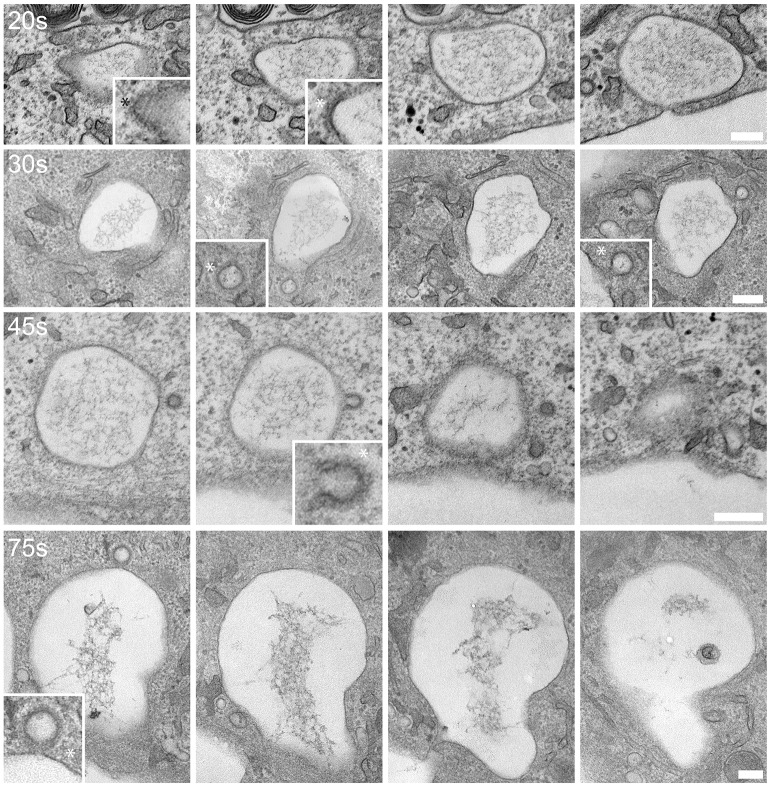
**Ultrastructure of compensatory endocytic events following WPB fusion.** HUVECs were co-transfected with mCherry–P-selectinLum and GFP-VWF before live-cell imaging and CLEM. All images shown are 70-nm-thick serial sections through individual fused WPBs fixed at different time points post fusion as shown at the top left of each image series. Asterisks indicate examples of compensatory structures. Inserts show zoom of compensatory structures. The earliest time that compensatory structures are noted is 20 s post fusion and all such structures are clathrin coated. Representative images are taken from *n*=6 experiments. Scale bars: 200 nm.

In total, we carried out such analyses in six separate experiments. Compensatory events (defined as endocytic budding profiles present on fused WPBs) were first seen 20 s after fusion, and fully invaginated endosomes were apparent as early as 30 s post fusion (Fig. 3). These early retrieval events took place whilst the exocytic pore was open and continued at later time points (60-75 s) as the pore closed. All events examined were clathrin coated and occurred directly on the exocytic structure. The compensatory endocytic structures also often contained electron-dense material that in most cases appeared to be near the membrane of the vesicle (Fig. 3). Fully-fused WPBs contained some VWF and there were apparent attachment sites between the internal VWF and the exocytic membrane. Note, endocytic events were not present on all exocytic structures (we quantified this more fully in fixed CLEM experiments; see below).

These results indicate that retrieval of WPB membrane, and presumably membrane proteins, begins very soon after fusion, and can occur while a pore (which can last more than 1 min) is open. Capacitance data indicates that, at least in some cases, the whole WPB membrane is retrieved intact from the plasma membrane after fusion (Zupancic et al., 2002). This, combined with our ultrastructural data, suggests a model whereby there is some immediate retrieval following WPB fusion, then the plasma membrane reseals and the internalised vacuolar structure is further ‘nibbled’ by smaller clathrin-coated buds to retrieve yet more membrane and integral membrane proteins.

### Low pH prevents unfurling of VWF

**Fig. 4.**
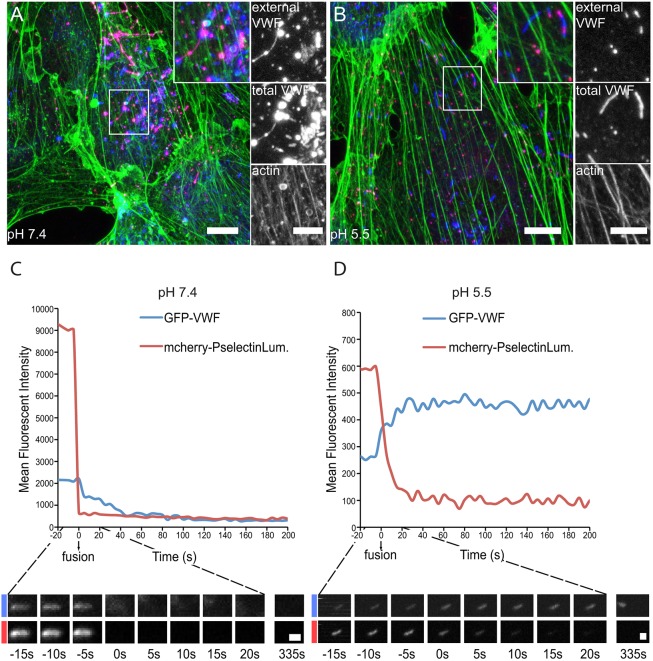
**Effect of low pH on WPB fusion.** (A,B) HUVECs stimulated with PMA (100 ng/ml) for 10 min in (A) pH 7.4 or (B) pH 5.5 medium. Cells are labelled for external VWF (magenta) total VWF (blue) and actin cytoskeleton (green). Images shown are maximum intensity projections. The boxed area is shown magnified in the inserts. (C,D) HUVECs were co-transfected with mCherry–P-selectinLum and GFP-VWF before PMA stimulation (100 ng/ml) at (C) pH 7.4 or (D) pH 5.5. Graphs indicate changes in mean fluorescent intensity 20 s before fusion and 200 s post fusion for the representative individual WPB shown below at each time point. At pH 7.4, WPBs collapse and lose content. At pH 5.5, fused WPBs lose the marker for fusion but retain VWF and stay rod shaped. Images show individual slices. Scale bars: 10 μm (A,B), 5 μm (inserts in A,B) and 1 μm (C,D).

The large stepwise decreases recorded by capacitance analysis suggest that pore sealing and bulk retrieval of WPB membrane is a general consequence of exocytosis of these huge granules (Zupancic et al., 2002). This should be sufficient to maintain cell size homeostasis, yet we observed a number of retrieval events that occur before pore closure (Fig. 3). This strongly suggests that there is an urgent requirement for the removal of membrane content introduced by exocytosis from the cell surface. To determine the relationship between pore closure and retrieval, we sought ways to limit the former by preventing the unfurling of VWF into strings. One way to do this is by minimising the pH change associated with exocytosis. When release of VWF occurs at lower pH, such as might occur during acidosis (pH 6.5), the rate of VWF unfurling is significantly slower and thus its release through the pore is delayed, blocking closure (Babich et al., 2009). The internal pH of WPB is 5.5 (Erent et al., 2007). We therefore reduced the pH in the HUVEC bathing medium to mirror this and stimulated exocytosis using PMA before labelling external and total VWF. At neutral pH, we saw collapsed WPBs releasing VWF as pronounced strings, as well as rod-shaped WPBs that are yet to fuse with the membrane (Fig. 4A). At pH 5.5, most of the WPBs remained rod-shaped (we did see some collapsed or more rounded WPBs but they were markedly less common) and no strings of VWF were seen (Fig. 4B). We also monitored fusion using live-cell imaging. At pH 7.4s we noted a rapid loss of mCherry–P-selectinLum and a transient increase in GFP-VWF intensity, followed by a more gradual diffusion away of the content as expected (Fig. 4C). During exocytosis at pH 5.5, however, mCherry–P-selectinLum exits more slowly and the GFP-VWF signal initially increases then remains largely the same for extended periods (Fig. 4D).

### VWF content release is required for normal compensatory endocytosis and WPB fusion

**Fig. 5.**
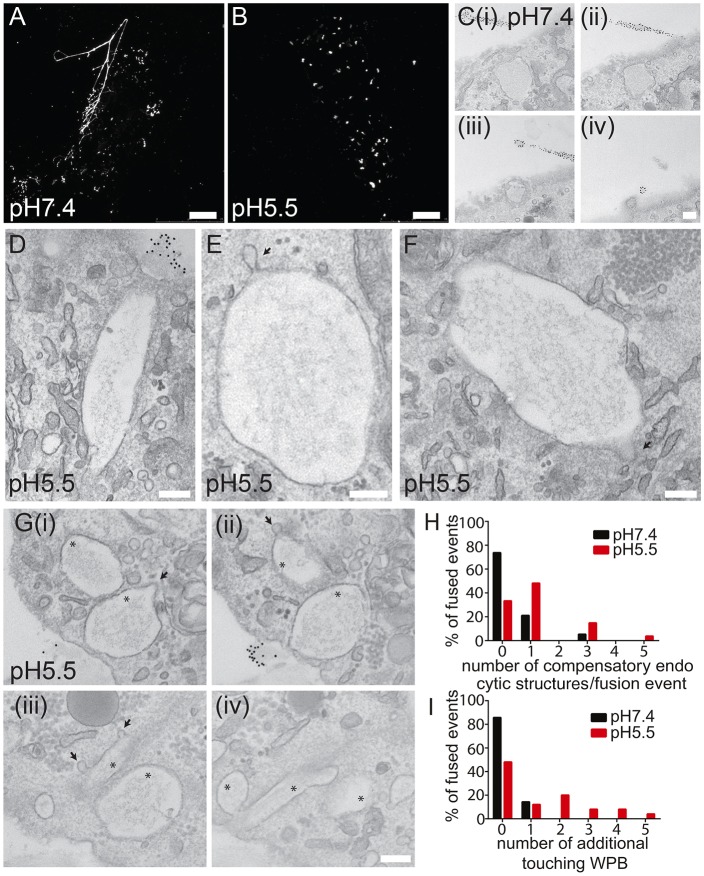
**Correlative electron microscopy of WPB fusion at low pH.** HUVECs were stimulated with PMA (100 ng/ml) for 10 min in pH 7.4 (A,C) or pH 5.5 (B,D-G) medium. Cells were then fixed and external VWF labelled with anti-VWF antibodies and a combination of Alexa Fluor 488 nm and 10 nm Protein-A–gold before imaging by confocal microscopy (A,B; maximum intensity projections) and preparation for CLEM (C-G, 70 nm serial sections). (A,C) At neutral pH, WPBs collapse and strings of VWF are clearly visible. (B,D-F) At pH 5.5, WPBs retain tubulated VWF and WPBs vary from completely collapsed to entirely rod shaped. All WPBs shown were fused as indicated by the presence of external gold labelling (not shown); arrows indicate compensatory endocytic structures. (G) Cumulative fusion events are apparent at pH 5.5. Arrows show compensatory endocytic structures, asterisks indicate fused collapsed WPBs. (H) Quantification of the distribution of compensatory endocytic profiles at pH 7.4 and pH 5.5. At pH 7.4, most profiles lack compensatory structures, but at pH 5.5, nearly 70% still have compensatory endocytic structures present. Data are pooled from *n*=4 experiments. (I) EM quantification of the prevalence of cumulative exocytosis at pH 5.5 and pH 7.4. TEM images in which fused (as evidenced by gold labelling) WPB membranes were clearly touching other collapsed or unfused WPBs were classed as cumulative. Data are pooled from *n*=4 experiments. Scale bar 10 μm (A,B), 200 nm (C-G).

To determine what happened to compensatory endocytosis at these unresolved fusion pores, we carried out CLEM of PMA-stimulated cells undergoing exocytosis at pH 7.4 (Fig. 5A,C) and at pH 5.5 (Fig. 5B,D-G). Following fixation, we labelled external VWF with 10 nm gold to demonstrate conclusively that VWF had been exocytosed, and then carried out serial sectioning. As we and others have previously found (Erent et al., 2007; Nightingale et al., 2011; Hannah et al., 2005), at pH 7.4, collapse of the WPB from a rod-shaped to a round structure is apparent, as is the release of VWF as strings (Fig. 5A,C). Fusion at pH 5.5 results in varying degrees of collapse, from structures that remain almost entirely rod-shaped (Fig. 5D), to slightly swollen fused structures (Fig. 5F) and WPBs that have actually collapsed (Fig. 5E). In all instances, at pH 5.5 we saw much more highly structured VWF that also showed some filamentous attachment to the organelle membrane.

When we quantified the number of compensatory endocytic structures on fused WPBs under each condition we noted an increase in events at lower pH (Fig. 5H). At pH 7.4, 25% of events seen by CLEM have compensatory profiles (between 1 and 3) whilst the remaining 75% lack obvious structures. In contrast, 70% of fused structures have up to five compensatory events at pH 5.5. This could indicate that either compensatory structures are taking longer to resolve at pH 5.5 or that more endocytic events are occurring.

Importantly, we also saw a change in the exocytic mode at pH 5.5, namely a marked increase in what appeared to be cumulative exocytosis, whereby a fused WPB acts as an exocytic site for a second-such organelle. In some situations, we saw up to four WPBs in close apposition with each other, such that their membranes are touching (Fig. 5G). Such apparent cumulative fusion was a relatively rare occurrence under control conditions with only 10% of fusing WPBs exhibiting some membrane-to-membrane contact with another WPB. In contrast, following a reduction in the pH of the bathing medium, this increased to more than 50% of events (Fig. 5I).

### WPB exocytic mode is influenced by VWF content release

**Fig. 6.**
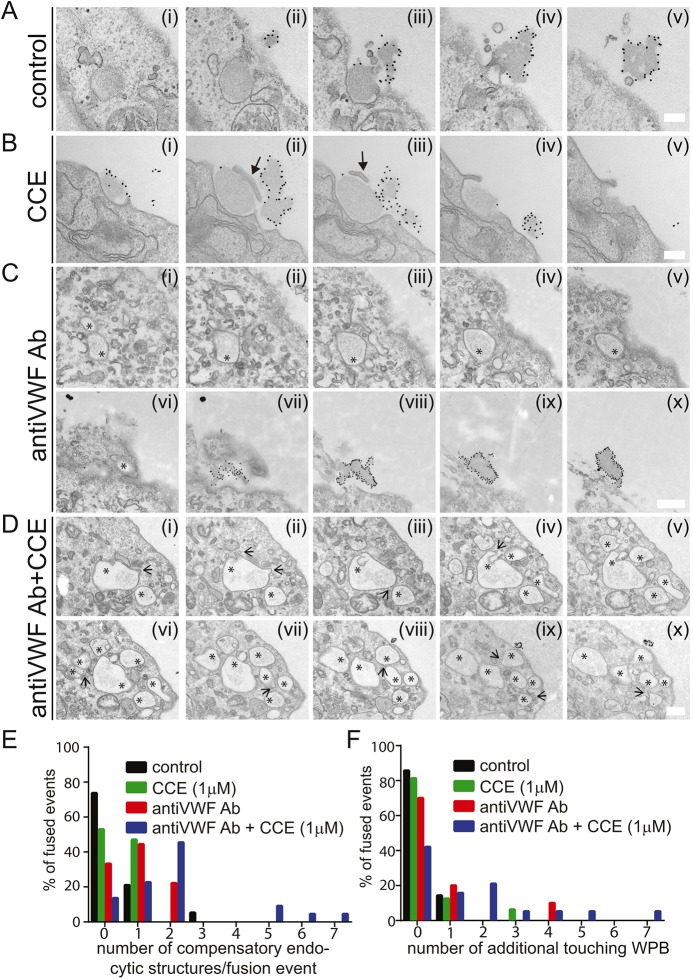
**Reduction in the rate of content release is correlated with increased compensatory endocytosis and cumulative exocytosis.** HUVECs were stimulated with PMA (100 ng/ml) for 10 min (A) and supplemented with (B) 1 μM cytochalasin E (CCE), (C) anti-VWF antibody (1 in 500) or (D) both agents together. Cells were then fixed and external VWF labelled with anti-VWF antibodies before preparation for TEM. Image series are from serial sectioning. Arrows with solid heads show pieces of membrane still covering the pore, open arrows show compensatory endocytic structures, asterisks indicate fused collapsed WPBs. (E) Quantification of the distribution of compensatory endocytic profiles following stimulation in control conditions and in the presence of CCE, antibody (Ab) or Ab+CCE. (F) EM quantification of the prevalence of cumulative exocytosis in the presence of CCE, Ab or Ab+CCE. TEM images in which fused (as evidenced by gold labelling) WPB membranes were clearly touching other collapsed or unfused WPBs were classed as in the process of cumulative fusion. Data are pooled from *n*=10 control, *n*=6 CCE and *n*=3 Ab+CCE experiments. Cumulative exocytosis is associated with an increase in compensatory exocytosis and is most prevalent when antibody is present in the medium and the actin ring is depolymerised. Scale bars: 200 nm (A,B), 500 nm (C,D).

To determine if the change in exocytic mode and the number of compensatory endocytic profiles was due to a failure to unfurl VWF tubules at low pH or a more general effect of delaying content release, we identified other approaches to inhibit the latter. We have previously demonstrated that an actomyosin ring is required for efficient expulsion of VWF following PMA stimulation (Nightingale et al., 2011). Inhibition of this mechanism can thus be used to prevent resolution of fusion at normal pH. Under control conditions, VWF was released from the fused WPBs and projected as strings above the plasma membrane in serial EM sections (Fig. 6A). However, in conditions where actin polymerisation was inhibited using cytochalasin E (CCE), we saw fused structures with pieces of membrane still covering parts of the open pore (Fig. 6B). We have previously shown that these structures can persist for over 300 s (Nightingale et al., 2011). When we analysed the number of compensatory vesicles present on these fused WPBs we saw only a small increase following CCE treatment (Fig. 6E). This had no effect on the rate of cumulative fusion (Fig. 6F). In fact, in some CCE-treated cells we saw multiple exit sites occurring in very close proximity with no evidence of cumulative fusion (Fig. S2). This demonstrates that the presence of a fused structure alone does not affect the exocytic mode.

As a third alternative approach to prevent VWF unfurling, we stimulated cells in the presence of an anti-VWF antibody in the bathing medium. Under these conditions, we noted an arrest of VWF near the exocytic site (as has been published previously by Knop et al., 2004) and no strings were formed. This method had a greater effect on the number of compensatory structures seen than CCE and a minor effect on cumulative fusion (Fig. 6C,E,F). The most marked effects were apparent when we combined the anti-VWF antibody and CCE (Fig. 6D). Here, we noted multiple compensatory endocytic profiles on almost all events (90%) (Fig. 6E) and a marked increase in cumulative fusion, similar to the effects of low pH (Fig. 6F). Generally, therefore, we noted a correlation between the number of compensatory endocytic structures and cumulative exocytosis. There was a threshold effect, with marked amounts of cumulative fusion only occurring when more than 70% of events had compensatory structures. This demonstrates that cumulative fusion is increased in situations where VWF cannot fully exit the cell and the pore is prevented from closing normally.

### Compensatory endocytosis is required for normal WPB fusion

The increase in frequency with which we observe compensatory structures by EM could be due to an increase in the number of events occurring or in the time it takes to resolve the structure and complete scission. To address this question, we interfered with the endocytic process directly. We know that compensatory endocytic events require clathrin from our EM experiments (Figs 2 and 3) and we could also see clathrin co-localising with the sites of VWF exocytosis (Fig. S3). We therefore chose to target this molecular aspect of the endocytic process. Blocking clathrin function on a long-term basis interferes with constitutive endocytosis and the formation of WPBs (Lui-Roberts et al., 2005), so we used the dynamin inhibitor Dynasore (Macia et al., 2006). To corroborate the effects of this treatment we also knocked down the likely adaptor protein AP-2 by transfecting siRNA targeting the AP-2 α-subunit (also known as AP2A1; Motley et al., 2003; Motley et al., 2006). Dynamin is required for both clathrin-dependent and some clathrin-independent pathways, and is necessary for fluid-phase HRP uptake (Holroyd et al., 2002) whereas AP-2 is required solely for clathrin-mediated endocytosis (Motley et al., 2003, 2006). Dynasore allows an acute inhibition of this process, whereas siRNA treatment required 3 or 4 days. AP-2α protein levels were reduced by 74% following siRNA treatment when compared to mock-treated cells, as determined by western blotting (Fig. S4A,B). We did see some co-localisation of dynamin II and external VWF by confocal microscopy, but perhaps due to the transient nature of dynamin function such events were relatively rare (Fig. S3).

**Fig. 7.**
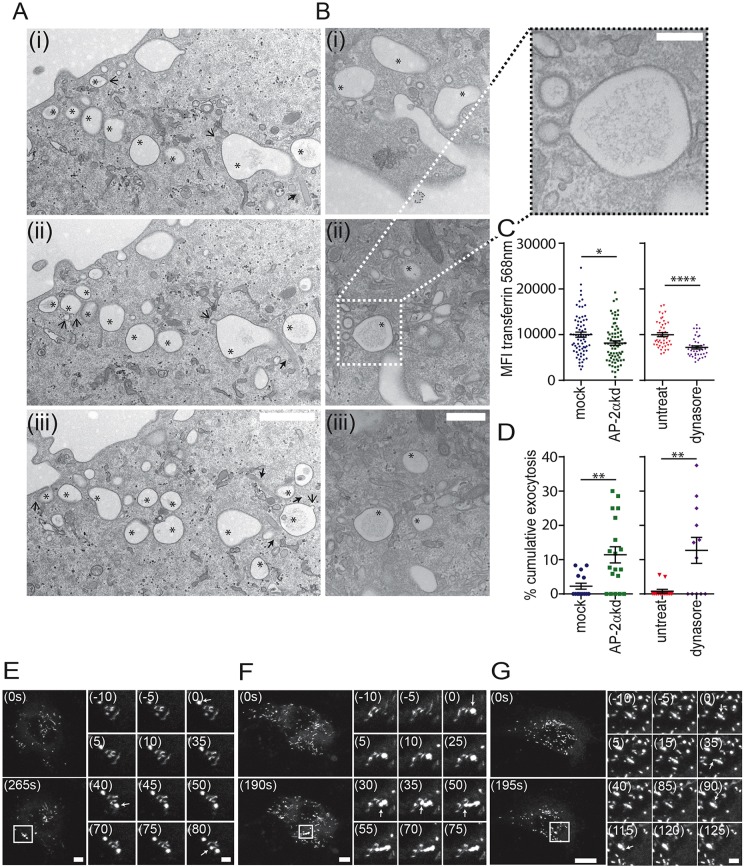
**Inhibition of compensatory endocytosis increases the likelihood of cumulative exocytosis.** (A-G) HUVECs were pre-incubated with control medium or 25 μM Dynasore-supplemented medium for 15 min before stimulation with 100 ng/ml PMA. (A,B) Cells were fixed after 10 min and prepared for TEM. Representative serial sections show multiple collapsed WPB (denoted by asterisks) with membranes touching projecting from the cell surface into the cytoplasm. Unfused WPBs (denoted by solid arrow heads) are also seen touching these collapsed structures. Open arrows show compensatory endocytic structures. The boxed area in Bii is shown at a higher magnification showing two clathrin-coated compensatory endocytic structures with wide necks. This likely reflects inhibited dynamin pinchase function. (C) Alexa Fluor 568-labelled transferrin was added to untreated, Dynasore-treated or AP-2α knockdown cells for 15 min. The mean fluorescence intensity of each cell over background was determined using ImageJ software (control, *n*=45 cells; Dynasore, *n*=40 cells; mock, *n*=69 cells; AP-2α, *n*=75 cells pooled from 4 experiments). (D-G) Control (E,F) or AP-2α knockdown (G) HUVECs were transfected with GFP-VWF and pre-incubated with (E,G) control medium or (F) 25 μM Dynasore-supplemented medium for 15 min, before stimulation with 100 ng/ml PMA and imaging for 10 min on a scanning confocal microscope. (D) Quantification of cumulative exocytosis in the presence and absence of 25 μM Dynasore (*n*=13 control; *n*=13 Dynasore-treated cells; *n*=15 mock-treated cells; and *n*=19 AP-2α knockdown cells from 4 experiments). Representative images of (E) untreated cells at 0 and 265 s, (F) Dynasore-treated cells at 0 and 190 s post stimulation are shown. (G) AP-2α knockdown cells at 0 and 195 s, with the time in the top left representing the time of the first fusion event. Arrows indicate point of WPB fusion. (E) In control cells, despite fusion events occurring in close proximity, and within 80 s, the sites remain separate. (F) Fusion events occurring in close proximity and within 75 s in Dynasore-treated or within 125 s in AP-2α knockdown cells merge in what appears to be cumulative exocytosis. Bars represent mean±s.e.m. *P<0.05, **P≤0.001, ****P<0.0001, Student's *t*-test. Scale bars: 1 μm (A), 500 nm (B), 200 nm (insert in B). Scale bar 10 μm (E-G), 2 μm (insets in E-G).

Treatment of HUVECs with 25 µM Dynasore or AP-2α siRNA blocked transferrin uptake as expected (Fig. 7C) and in the case of dynamin treatment caused a marked change in the exocytic mode, as revealed by EM (Fig. 7A,B). We often noted chains of large mostly empty vacuoles in close apposition, stretching from the plasma membrane into the cytoplasm. These often contained some remnants of VWF cargo. We also noted the presence of unfused WPBs touching some of these vacuoles (Fig. 7A,B). As expected, many more clathrin-coated endocytic profiles were apparent, and these often exhibited wide necks, presumably due to the inhibited pinchase function of dynamin (Fig. 7B). Definition and therefore accurate quantification of exactly which vacuoles unambiguously represented previously fused WPBs was difficult because not all such vacuoles contained obvious VWF content (previous analysis was possible due to arrested VWF secretion or by using gold-conjugated anti-VWF labelling at the cell surface). To corroborate the EM, we also carried out live-cell imaging of the exocytosis of WPBs labelled with VWF-GFP in the presence or absence of 25 µM Dynasore or following treatment with siRNA targeting AP-2α. In control cells, WPBs fused in close proximity without merging (Fig. 7D,E). Following Dynasore treatment (Fig. 7D,F) or AP-2α siRNA treatment (Fig. 7D,G) a greater proportion of exocytic events merged into one another, suggesting cumulative fusion. Dynasore treatment did not affect the rate of granule collapse or the extent of lingering kiss fusion. The difference in cumulative fusion quantified by live-cell imaging although significant was lower than that obtained using EM quantification. This is likely because of an underestimation of cumulative fusion events due to the release of VWF-GFP (it is impossible to tell if fused structures merge if the content has already been released from one of these structures). These data support the conclusion that the increase in cumulative exocytosis shown by blocking content release is due to an inhibition of compensatory endocytosis.

### Cumulative fusion of WPBs results in improper release of VWF strings

**Fig. 8.**
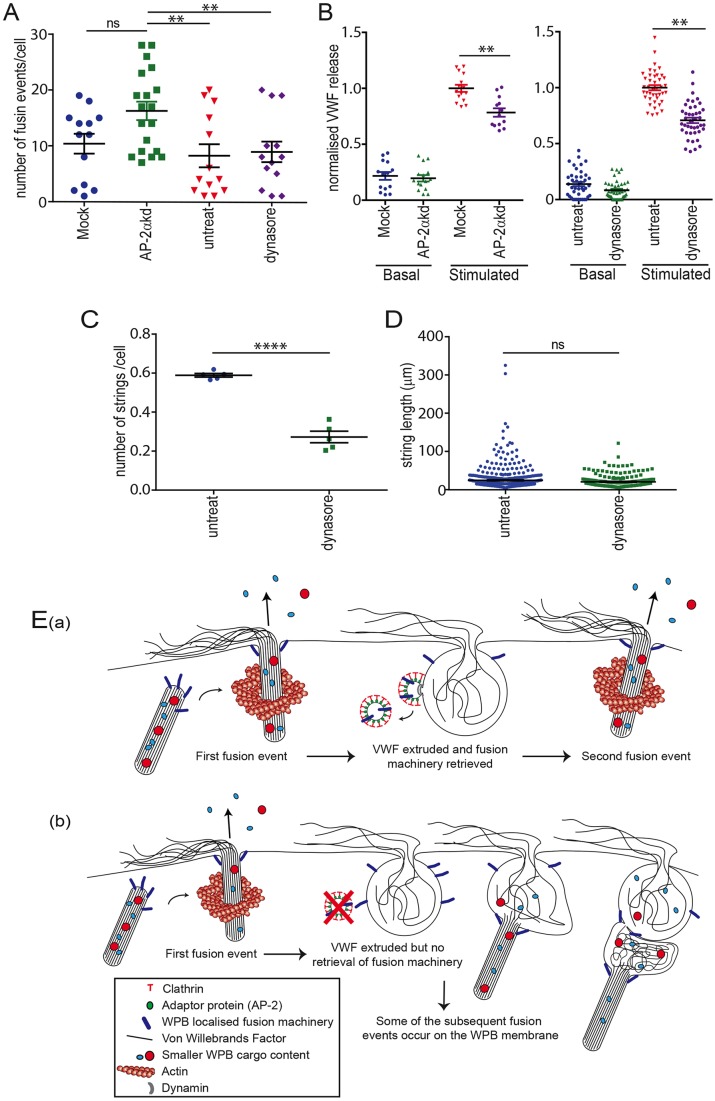
**Cumulative fusion of WPBs results in improper release of VWF strings.** (A,B) Control, mock, 25 μM Dynasore-treated or AP-2α knockdown HUVECs were stimulated with 100 ng/ml PMA. (A) The number of fusion events was monitored over a 10 min period by scanning confocal microscopy. There was no significant difference in the extent of fusion between Dynasore-treated and untreated cells (*n*=13 control and drug-treated cells from 4 experiments). A significant difference in the amount of fusion was noted in AP-2α knockdown compared with mock, control and Dynasore-treated cells. Bars represent mean±s.e.m. **P≤0.01; ns, not significant, one-way ANOVA with Kruskall-Wallis post-test. (B) Treated, Dynasore-treated mock and AP-2α knockdown cells were incubated in the presence or absence of PMA (100 ng/ml) and the amount of VWF release determined by ELISA as a fraction of total VWF. Data are normalised to control and there is a small but significant inhibition of VWF release noted. Bars represent mean±s.e.m. **P<0.01, Student's *t*-test. Data represent six individual measurements from *n*=7 Dynasore or *n*=5 AP-2α knockdown experiments. (C,D) Dynasore-treated or untreated cells were stimulated under flow with 100 ng/ml PMA and (C) the number and (D) length of VWF strings determined. ****P<0.0001, Student's *t*-test; ns, not significant. Representative data shown from *n*=3 experiments. (E) Model of the mechanism and function of compensatory endocytosis in endothelial cells. (a) WPBs dock and fuse with the plasma membrane, releasing VWF strings as well as smaller soluble and membrane-bound cargo. The majority of WPB exocytic events do not result in full fusion (i.e. total collapse of the organelle into the plasma membrane). Instead, fused empty WPBs persist as distinct structures on which clathrin-coated budding vesicles assemble as early as 20 s post exocytosis. The formation of AP-2-mediated clathrin-coated vesicles occurs irrespective of whether the pore is open and such events serve to retrieve fusion machinery in a dynamin- and AP-2-dependent manner. The fusion pores close after ∼60-75 s whilst clathrin-mediated budding continues. (b) When clathrin-mediated endocytosis is inhibited either via inhibition of AP-2 or dynamin, this causes WPB fusion machinery to build up on the post-fusion granule membrane. This makes such sites favourable for WPB fusion causing cumulative exocytosis. VWF released from such cumulative events cannot unfurl properly under flow and becomes tangled, resulting in shorter strings.

To investigate whether the switch toward cumulative fusion would have functional consequences, we analysed the release of VWF and the formation of platelet-catching VWF strings under different conditions. Live-cell imaging indicated no difference in the number of WPB fusion events per cell following dynamin inhibition (Fig. 8A), yet we noted a small but significant decrease in the amount of VWF secreted (∼29.2±2.4%, Fig. 8B). The depletion of AP-2α by siRNA resulted in a similar reduction in the fraction of total cellular VWF released (∼21.2±4.0%, Fig. 8B), yet caused an overall increase in the number of fusion events. Further analysis revealed this was due to a significant increase in WPB number (Fig. S4C) and VWF levels (Fig. S4D,E) in AP-2 knockdown cells. This most likely reflects a long term-effect of AP-2 depletion (see discussion below). Despite this increase in WPB number, we still see the same effects on cumulative fusion and the fraction of VWF released.

Analysis of VWF string number and length under flow demonstrated that there was a marked reduction (more than 2-fold) on the number of strings/cell following Dynasore treatment (Fig. 8C) with little overall effect on string length (Fig. 8D) (apart from perhaps for very long VWF strings). This indicates that cumulative exocytosis results in aberrant release of VWF, most probably due to its inefficient release as tangled strings. Interpretation of the effect of the longer-term AP-2 knockdown on string length was impossible because of the increase in WPB number relative to that in the mock-transfected control (Fig. S4C-E), so that we were unable to separate the effects on string length of tangled VWF from the effects on WPB number.

## DISCUSSION

The work described here demonstrates that compensatory endocytosis in endothelial cells is a major endocytic route and is likely to significantly influence a normal haemostatic response. By measuring the incorporation of fluid-phase HRP in the presence or absence of WPBs we have determined that approximately one-third of endocytic uptake by volume – as measured by HRP uptake – following PMA stimulation is internalised by compensatory pathways (Fig. 1). Essentially all of this stimulated endocytosis was due to WPB exocytosis, with a minimal contribution via fusion of other organelles such as lysosomes. We also provide the first ultrastructural characterisation of compensatory endocytosis in endothelial cells. We show that the majority of WPB exocytic events, not just the minority comprising lingering kiss (endothelial kiss-and-run; Babich et al., 2008) events, do not result in full fusion, i.e. total collapse of the organelle into the plasma membrane (Figs 2 and 3). Instead, fused empty WPBs persist as distinct structures on which clathrin-coated budding vesicles assemble as early as 20 s post exocytosis. The formation of clathrin-coated vesicles occurs irrespective of whether the pore is open and they often contain membrane-associated electron-dense cargo. Fusion pores close after ∼60-75 s whilst clathrin-mediated budding continues. This model of retrieval is in line with the cavicapture events seen in chromaffin cells (Barg and Machado, 2008; Houy et al., 2013), i.e. recapture of the profile followed by continued CME ‘nibbling’ to break down the structure. Such a model agrees with published analysis of capacitance changes during regulated release in endothelial cells (Zupancic et al., 2002). Over extended measurements, symmetrical distribution of positive (exocytic) and negative (endocytic) event amplitudes have been characterised, indicating that a similar amount of membrane is added to the plasma membrane as is retrieved. The proportion of events resolved between 0.5 and 110 fF account for ∼33% of the capacitance change and this is in line with the changes we note in HRP uptake on PMA stimulation. Our ultrastructural experiments only monitor events that occur directly on the exocytic structure and we are only addressing one stimulus. As such, we cannot rule out the presence of additional compensatory endocytosis at discrete sites including events such as ultrafast endocytosis and activity-dependent bulk endocytosis (Soykan et al., 2016) in this study. Given that the rate of WPB fusion (relative to synaptic vesicle release) is relatively slow and the exocytic capacitance changes approximately mirror endocytic changes, their contributions are likely to be relatively minor.

We did not note any clathrin-coated pits on mature WPBs (typified by electron-dense tubules of VWF) or on fusion events earlier than 20 s post fusion. Indicating that such events occur post exocytosis. Clathrin-coated buds have been noted on immature (electron-lucent) WPBs near the TGN, presumably reflecting the retrieval of mis-sorted granule components (Zenner et al., 2007).

The function of compensatory endocytosis in endothelial cells is likely to be very different from other systems, such as in neurons, where the primary purpose is to quickly replenish synaptic vesicles to maintain exocytic output. Functional WPBs can only be formed at the TGN (Lenting et al., 2015; Lui-Roberts et al., 2005; Zenner et al., 2007; Lui-Roberts et al., 2008), thus any direct replenishment by compensatory endocytosis would be impossible. Also, we have shown that direct inhibition of endocytosis using dynamin inhibitors had little effect on the extent of WPB fusion (Fig. 8A). Despite this, the assembly of clathrin-coated structures directly on the WPB membrane so soon after fusion does suggest a requirement for rapid retrieval of certain proteins or lipids (Fig. 3). Our data suggest a link between content release, compensatory endocytosis and the exocytic mode. We manipulated conditions to drive incomplete VWF release, using either bathing medium at low pH, the presence of anti-cargo antibody in the bathing medium, or inhibition of actin remodelling. These treatments all resulted in an increased number of compensatory profiles. All of these approaches have their own particular limitations (more gross effects of the pH change, global effects on the cytoskeleton etc.), but despite these differing limitations, the same consensus effect is apparent i.e. a change in exocytic mode is coupled to a change in clathrin-coated structures. This change in exocytic mode is also phenocopied by more-specific approaches; the acute inhibition of endocytosis using the dynamin inhibitor Dynasore or the longer-term siRNA-mediated depletion of AP-2α, together indicating that this is an endocytosis-specific phenomenon (Fig. 7).

Importantly, the changes in content release that drive the increase in compensatory structures also produced a change in exocytic mode, with cumulative fusion events becoming markedly more common. We noted a correlation between the number of compensatory structures seen and the incidence of cumulative fusion (Figs 5 and 6); the greatest effects on cumulative exocytosis occurred when compensatory structures were present on more than 70% of events. This suggests there may be some sort of threshold effect involved. It is unclear precisely how inhibition of VWF content release influences compensatory endocytosis. Perhaps this is simply a physical constraint due to difficulty retrieving membrane from a structure still containing content or perhaps resealing of the granule is necessary for efficient endocytosis. Alternatively, it might be that signalling events are triggered following content clearance that acts upon the endocytic machinery. We also noted an increase in the total amount of VWF within AP-2α knockdown cells by western blot (Fig. S4D) and ELISA (Fig. S4E). The reasons for this increase are unclear. Clathrin and AP-1 are required for WPB formation (Lui-Roberts et al., 2005) and long-term inhibition of AP-2 may therefore result in increased pools of unused clathrin and consequently an increase in WPB number. Alternatively, inhibition of AP-2 might have interfered with a signal required to limit WPB formation or control the basal release of WPB.

Our data indicates the change in exocytic mode is due to compensatory events that are arrested, or resolve more slowly. We propose that this affects exocytosis by causing a failure to release for re-distribution membrane proteins required for exocytosis (see model Fig. 8E). This leads to an accumulation of the fusion machinery and associated components at release sites, which then drives cumulative exocytosis. In neurons compensatory endocytosis has been shown to play a role in clearing exocytic machinery from release sites (Soykan et al., 2016; Hua et al., 2013; Jung et al., 2015). We hypothesise that this is also the purpose of the early clathrin-mediated events on fused WPB membranes. Failure to resolve compensatory endocytic events increases cumulative fusion to over 50% (from TEM characterisation) (Figs 4–7). We have yet to determine the integral membrane proteins that are retrieved from WPBs (this research is in progress). During neurotransmission, the molecules retrieved include vSNAREs, synaptotagmin and synaptophysin (Soykan et al., 2016). In endothelial cells, the only analogous characterised membrane-bound machinery shown to be localised to WPBs are the v-SNARE proteins VAMP-3 and -8 (Pulido et al., 2011). Interestingly, sequential fusion of granules in pancreatic acinar cells has shown to be dependent on the retrieval of VAMP-8. Mice lacking this SNARE exhibit a specific reduction in secondary fusion events (granule to granule) with no difference in the fusion of granules with the plasma membrane (Behrendorff et al., 2011). Other factors that are likely to play a role in the amount of cumulative fusion are the number of WPBs and the rate of exocytosis. Live-cell imaging of WPB exocytosis following dynamin inhibition demonstrated that cumulative exocytosis was most common when the fusion rate was high.

Control of the exocytic mode is important to allow the release of a normal complement of VWF strings under flow (Fig. 8). Acute inhibition of compensatory endocytosis does not affect the extent of WPB fusion (Fig. 8A) but does result in a small inhibition of VWF release (Fig. 8B). However, the effect on the number of VWF strings is much more marked (Fig. 8C), indicating that cumulative fusion is significantly less effective for releasing strings of VWF under flow. This mode of release results in a greater amount of VWF being released through one pore; the strings are therefore much more likely to be tangled and the shear force would presumably act less efficiently to pull strings out at the cell surface. This is likely to have a negative effect on platelet recruitment, and thus haemostatic function.

Generally, and in various cell types, two different mechanisms of bulk exocytosis can occur (Pickett and Edwardson, 2006). In mast cells, compound exocytosis results from granules fusing with each other before fusing with the plasma membrane (Alvarez de Toledo and Fernandez, 1990) whereas during cumulative exocytosis, as occurs in the pancreatic acinar, granules fuse with others that have already exocytosed. Both mechanisms allow for focused content release at a very small region of the plasma membrane (Nemoto et al., 2001; Thorn and Parker, 2005). Our experiments at low pH strongly suggest a cumulative rather than a compound mode of content release for WPBs. The WPBs are more rod-shaped at low pH even after fusion and we can tell that this fusion is sequential as the most collapsed structure is always adjacent to the plasma membrane and the WPBs fusing with this become progressively more rod-shaped deeper inside the cell. Cumulative fusion events in endothelial cells have been suggested to occur previously by monitoring the transfer of overexpressed CD63-GFP from sequentially fusing WPBs (Kiskin et al., 2014), but such events have never been previously characterised at the TEM level nor quantified. Compound exocytosis has also been reported to occur in endothelial cells by the formation of a pre-fusion structure termed a secretory pod (Valentijn et al., 2010). This is thought to form by fusion of WPBs with each other before subsequent fusion with the cell surface. Interestingly these structures also exhibit apparent compensatory structures. If such a model is correct it would require a pH change as the WPBs that form the pod are collapsed and contain disordered VWF. It is not yet resolved as to how this occurs. Capacitance analysis of WPBs undergoing stimulation does to some extent support compound exocytosis, as steps that represent more than a single WPB have been noted, although infrequently (Zupancic et al., 2002). In our experiments we noted that the presence of antibody in the bathing medium alters exocytic mode. Given that the initial characterisation of the secretory pod was carried out in medium with anti-VWF antibody to capture and label exocytic sites, we suggest that this might have affected the interpretation of their results (Valentijn et al., 2010). An alternative model for pod formation supported by our data would be cumulative fusion of WPBs with resealed lingering kiss structures. This would result in large internal structures and, because VWF release is compromised, these would also be decorated with more compensatory endocytic structures. The authors of the paper describing secretory pods highlight the possibility of this model in a more recent publication (Mourik et al., 2013).

We also noted attachment of VWF to the sides of exocytic structures. This has been seen before (Valentijn et al., 2010) and its purpose is as yet unclear. VWF attachment occurs at both pH 5.5 and pH 7.4, suggesting that it is present before the tubules unfurl and the granule collapses. It may therefore represent a means to anchor strings to support orderly unravelling of content. Interestingly, in immature WPBs, the VWF tubules are clearly spaced away from each other and especially from the membrane along the longitudinal axis of the organelle, whereas inter-tubule structures can occasionally be seen, and the tubules appear to touch the limiting membrane at the organelle tips. Whether the attachment sites for VWF on the fused structure involve the same elements is not yet clear.

Overall, we provide the first characterisation of compensatory endocytosis in endothelial cells using biochemical and novel ultrastructural techniques. By fully characterising this pathway, we were able to demonstrate that it influences the exocytic mode of WPBs. This is a novel function for this poorly understood process in endothelial cells, and is particularly important for VWF exocytosis whereby orderly release of untangled VWF strings is a prerequisite for effective platelet recruitment and thus haemostasis.

## MATERIALS AND METHODS

### Cell culture and transfection

Human umbilical vein endothelial cells (HUVECs, Promocell, Heidelberg, Germany) were cultured as previously described (Michaux et al., 2006). Plasmid transfections were performed by nucleofection (Nucleofector II, programme U-001, Amaxa Biosystems, Gaithersburg, MD) using 2-5 μg DNA. GFP-VWF (Romani de Wit et al. 2003) was a gift from Jan Voorberg and Jan Aart Van Mourik (Sanquin Research Laboratory, Amsterdam, Netherlands). The synthesis of C-terminal-tagged mCherry–P-selectinLum fusion construct was described in Nightingale et al. (2011). Mock, VWF and AP-2α siRNA transfections were performed as in Ferraro et al. (2016) using firefly luciferase siRNA, 5′-CGUACGCGGAAUACUUCG-3′; VWF siRNA, 5′-GGGCUCGAGUGUACCAAAATT-3′; and AP-2α siRNA, 5′-AAGAGCAUGUGCACGCUGGCCA (Motley et al., 2003) (Qiagen).

### Antibodies and reagents for immunofluorescence

Rabbit anti-VWF was purchased from DAKO (cat. no. A0082; 1:10,000). Sheep anti-VWF (cat. no. AHP002; 1:10,000) and sheep anti-TGN46 (cat. no. AHP500; 1:200) were purchased from BIORAD. Sheep polyclonal anti-P-selectin was from R&D systems (cat. no. AF137; 1:100), mouse monoclonal anti-EEA1 (clone 14, cat. no. 610457; 1:200) and mouse anti-adaptin α (cat. no. 610502; 1:1000) was from BD Pharmingen, and mouse anti-clathrin light chain (CON.1, cat. no. AB9884; 1:100) and mouse anti-tubulin were from Sigma.

### HRP assays

HUVECs were incubated with 1 mg/ml HRP (Sigma) with or without 100 ng/ml phorbol 12-myristate 13-acetate (PMA) for 15 min at 37°C, then washed thoroughly on ice and fixed in 4% formaldehyde (PFA, Polysciences, Edenkoben, Germany). HRP was visualised by fluorescein-tagged tyramide signal amplification; TSA-fluorescein (Perkin-Elmer) was diluted 1:150 in the provided diluent and incubated with coverslips for 2 min before stopping the reaction with a 30 s incubation with 0.2% (w/v) sodium azide. Cells were then washed with PBS, antibody-labelled and imaged as above. For co-localisation with transferrin, 20 μg/ml Alexa Fluor 568-conjugated transferrin (Life Technologies, Paisley, UK) was included in the incubation medium. For quantification, ten fields of view were taken at random per experiment and HRP-positive objects quantified using ImageJ software; background subtraction was performed using a rolling ball algorithm (2 pixels) and a threshold manually applied prior to performing particle analysis to count objects over 0.1 μm.

### Immunofluorescence staining

Fixation and staining proceeded as in Lui-Roberts et al. (2005). Fixed cell images were taken on a Leica SPE scanning confocal microscope system with a 63× objective (NA 1.3) as confocal *z*-stacks with 0.5 μm step size. Acquisition was performed using LAS-AF software with a 1024×1024 pixel resolution, 3-4 frame average and 1× zoom. To image clathrin recruitment, confluent HUVECs were stimulated with 100 ng/ml PMA for 5 min then fixed in 4% paraformaldehyde (PFA) and stained for external VWF prior to permeabilising cells and staining for total VWF and clathrin. Cells were imaged as cross-sectional confocal *y*-stacks with a 0.5 μm step size on a Leica SPE scanning confocal microscope system as above. For fixation, following changes in pH, cells were cultured as before but stimulated in serum-free medium buffered with either 0.1 M HEPES or 0.1 M MES. Cells were fixed at 37°C in 4% methanol-free formaldehyde (TAAB Laboratories Equipment) in cytoskeleton buffer (10 mM MES, pH 6.1, 3 mM MgCl2, 138 mM KCl, and 2 mM EGTA) with 0.32 M sucrose and then permeabilised in 0.5% Triton X-100 (Sigma) and incubated with Alexa Fluor 488-conjugated Phalloidin (Life Technologies) and a relevant primary antibody.

### Western blotting

Proteins were separated by SDS-PAGE, transferred to Whatman nitrocellulose membranes (PerkinElmer), and then probed with primary antibody followed by the appropriate HRP-conjugated secondary antibody (1:5000) purchased from Jackson ImmunoResearch Laboratories (West Grove, PA, USA).

### Imaging live dextran uptake

Transfected cells were seeded onto eight-well LabTek sterile borosilicate coverglass chamber slides (Life Technologies). After 24 h, chambers were rinsed with Ringer's solution (140 mM NaCl, 5 mM KCl, 1.8 mM CaCl_2_, 2 mM MgCl_2_, 10 mM glucose, 20 mM HEPES-NaOH, pH 7.4) then mounted on a Leica TCS SP5 inverted microscope on a heated stage set at 37°C. Cells were stimulated with 100 ng/ml PMA in the presence of 1 mg/ml dextran-tetramethyl-Rhodamine (Life Technologies) and immediately imaged through a 63× oil immersion lens (NA 1.4) using an 8000 Hz high-resonance scanner. Single confocal plane images (1024×1024 pixels) were taken every 5-10 s for 3-5 min using a single line and frame average.

### Live-cell imaging

For live-cell imaging, knockdown and untreated cells were transfected with GFP-VWF and mCherry–P-selectinLum onto a glass-bottom dish or, for live-cell CLEM, a gridded coverslip-bottom dish (in situations where exocytosis from knockdown cells was being monitored, 300 pmol AP-2α siRNA was also included during the transfection). Imaging proceeded using a 100× oil immersion lens (NA 1.4) on a spinning-disk system (UltraVIEW VoX; Perkin-Elmer) mounted on an inverted microscope (TiE; Nikon, Kingston Upon Thames, UK) with an EM charge-couple device camera (512×512 pixels; C9100-13; Hamamatsu Photonics, Welwyn Garden City, UK) and 488 and 568 solid-state lasers. *Z*-stacks were acquired every 5 s using a Piezo (NanoSanZ; Prior Scientific, Cambridge, UK) with a step size of 0.4-0.5 μm, comprising 9-14 images with an exposure of 30 ms at either pH 7.4, pH 5.5 and in the presence or absence of 25 μM Dynasore (Sigma).

### Correlative EM

Exocytic site labelling assays were carried out using a modified method described by Knop et al. (Knop et al., 2004). Cells were cultured on gridded coverslip-bottom dishes (MatTek, Bratislava, Slovak Republic) and 24 h later incubated for 10 min in the presence of rabbit anti-VWF with PMA (100 ng/ml) either in the presence or absence of 1 μM cytochalasin E (CCE) or in the presence or absence of Dynasore (25 μM) at pH 7.4 or at pH 5.5 (see IF protocol above, all from Sigma). In experiments in which CCE was used, the cells were pretreated for 15 min prior to stimulation. CLEM was carried out as in Nightingale et al. (2011). For live-cell imaging, CLEM cells were transfected and imaged as above but beforehand the cell to be imaged was localised and DIC images acquired using 10× air (NA 0.3), 20× air (NA 0.7) and 40× oil (NA 0.75-1.25) objectives. At arbitrary time points, 4× fix was added to the cells and images were acquired post fixation to confirm efficacy. The remainder of the TEM preparation was carried out as conventional CLEM.

### Quantification of compensatory endocytic profiles by electron microscopy

Serial sections were arranged in order. Fused WPBs were denoted by demonstrable loss of the mCherry–P-selectinLum (in the case of correlative time-lapse imaging to electron microscopy) or the presence of gold-labelled anti-VWF antibody at the cell surface and the presence of a collapsed structure (in non-correlative and correlative microscopy). Any ambiguous structures were excluded from the analysis. To be denoted as bona fide compensatory endocytic structures, the budding vesicles had to have a demonstrable connection to the fused structure in at least one section. Tubular membrane structures such as endoplasmic reticulum (which are also often found near fusing WPBs) were excluded as they occur in more than 1 or 2 sections and lack a clathrin coat.

### String analysis

HUVECs were seeded onto µ-slides (Ibidi, Munich, Germany) with a 5-mm-wide channel 24 h before experiments. HUVECs were pre-treated with 25 µM Dynasore for 15 min or left untreated and slides subsequently attached to a syringe pump (Harvard Apparatus, Holliston, MA, USA) to draw fluid over the cells at a constant wall shear stress of 0.25 MPa (2.5 dynes/cm^2^). Cells were maintained at 37°C and were perfused with HBSS buffer (Hank's balanced salt solution containing Ca^2+^, Mg^2+^ and 0.2% BSA; Life Technologies) for 2 min to remove debris before being stimulated with PMA (100 ng/ml) in the presence or absence of Dynasore for 5 min in HBSS buffer (as above). Cells were fixed with 4% PFA under decreasing rates of flow for 10 min until static and left in PFA for a further 10 min. Samples were prepared for immunofluorescence without permeabilisation, and stained for surface-bound VWF via incubation with rabbit anti-VWF (DAKO, cat. no. A0082; 1:10,000) followed by Alexa Fluor 488-conjugated anti-rabbit secondary antibody and stained for DNA with Hoechst 33342 (Life Technologies). VWF strings were imaged by confocal microscopy (Leica TCS SPE) using a 40× (NA 1.15) oil objective. Maximum intensity projections were generated of confocal stacks in Fiji, which were used to manually count VWF strings.
